# Data for a comprehensive map and functional annotation of the human cerebrospinal fluid proteome

**DOI:** 10.1016/j.dib.2015.02.004

**Published:** 2015-02-20

**Authors:** Yang Zhang, Zhengguang Guo, Lili Zou, Yehong Yang, Liwei Zhang, Nan Ji, Chen Shao, Yajie Wang, Wei Sun

**Affiliations:** a Department of Neurosurgery/China National Clinical Research Center for Neurological Diseases, Beijing Tiantan Hospital, Capital Medical University, 6 Tian Tan Xi Li, Beijing 100050, China; b Core Facility of Instrument, Institute of Basic Medical Sciences Chinese Academy of Medical Sciences, School of Basic Medicine, Peking Union Medical College, 5 Dong Dan San Tiao, Beijing 100005, China; c National Key Laboratory of Medical Molecular Biology, Department of Physiology and Pathophysiology, Institute of Basic Medical Sciences Chinese Academy of Medical Sciences, School of Basic Medicine, Peking Union Medical College, 5 Dong Dan San Tiao, Beijing 100005, China; d Core Laboratory for Clinical Medical Research, Beijing Tiantan Hospital, Capital Medical University, 6 Tian Tan Xi Li, Beijing 100050, China; e Department of Clinical Laboratory Diagnosis, Beijing Tiantan Hospital, Capital Medical University, 6 Tian Tan Xi Li, Beijing 100050, China

## Abstract

Knowledge about the normal human cerebrospinal fluid (CSF) proteome serves as a baseline reference for CSF biomarker discovery and provides insight into CSF physiology. In this study, high-pH reverse-phase liquid chromatography (hp-RPLC) was first integrated with a TripleTOF 5600 mass spectrometer to comprehensively profile the normal CSF proteome. A total of 49,836 unique peptides and 3256 non-redundant proteins were identified. To obtain high-confidence results, 2513 proteins with at least 2 unique peptides were further selected as bona fide CSF proteins. Nearly 30% of the identified CSF proteins have not been previously reported in the normal CSF proteome. More than 25% of the CSF proteins were components of CNS cell microenvironments, and network analyses indicated their roles in the pathogenesis of neurological diseases. The top canonical pathway in which the CSF proteins participated was axon guidance signaling. More than one-third of the CSF proteins (788 proteins) were related to neurological diseases, and these proteins constitute potential CSF biomarker candidates. The mapping results can be freely downloaded at http://122.70.220.102:8088/csf/, which can be used to navigate the CSF proteome. For more information about the data, please refer to the related original article [1], which has been recently accepted by Journal of Proteomics.

**Table:** 

Subject area	*Biology*

More specific subject area	*Proteomics*
Type of data	*Tables*
How data was acquired	*Instruments including Waters Acquity nano-UPLC system, AB SCIEX Triple TOF* 5600 *system*
Data format	*Analyzed*
Experimental factors	*Protein samples were reduced with* 10 mM *DTT, alkylated with* 55 mM *iodoacetamide, digested using sequencing-grade modified trypsin.*
Experimental features	*CSF pooled from* 14 *individuals* (7 *women and* 7 *men*) *was subjected to the depletion of* 14 *high-abundance proteins with an immunoaffinity column. The flow-through proteins, bound proteins, and original proteins were collected separately, digested, and then separated into* 30 *fractions each by high-pH RPLC. Total* 90 *fractions were subjected to nano-RPLC-MS*/*MS analysis.*
Data source location	*Beijing, China*
Data accessibility	*The data in the data in brief, in the related original article*[1]*and at*http://122.70.220.102:8088/csf/*can be freely downloaded.*

**Value of the data**

• This study identified the largest high-confidence dataset of the human CSF proteome.
• Some CSF proteins׳ abundances are quantified by the iBAQ method.
• High proportion of the CSF proteins is microenvironment components of CNS cells.
• High proportion of the CSF proteins participate in neurocyte connectivity.
• A large part of CSF proteins are biomarker candidates of neurological diseases.

## 1 Data, experimental design, materials and methods

### 1.1 Data

Table 1 lists all the CSF proteins with at least 2 unique peptide identifications. Table 2 lists the proteins and their abundances, which were quantified by the iBAQ [2] method. Table 3 lists the CSF proteins that participate in the axon guidance signaling pathway. Table 4 lists the CSF proteins involved in neurological diseases.

### 1.2 Experimental design

CSF pooled from 14 patients (7 women and 7 men) who received spinal anesthesia before non-neurological operations was subjected to a global proteomic analysis. The pooled sample was first depleted of 14 high-abundance proteins with an immunoaffinity column. The flow-through proteins, bound proteins, and original proteins (extracted from the CSF samples that were not subjected to immunoaffinity depletion) were collected separately, digested according to the filter-aided sample preparation method [3], and then separated into 30 fractions each by high-pH RPLC. Each fraction was then subjected to proteomic analysis by nano-RPLC-MS/MS. In total, 90 LC-MS/MS runs were performed on the 90 fractions from the pooled CSF samples, and the resulting data were used to produce a comprehensive map of the human CSF proteome.

### 1.3 Materials and methods

#### 1.3.1 Apparatuses

A TripleTOF 5600 mass spectrometer from AB Sciex (Framingham, MA, USA) and an ACQUITY UPLC system from Waters (Milford, MA, USA) were used.

#### 1.3.2 Reagents

Deionized water from a MilliQ RG ultrapure water system (Millipore, Bedford, MA, USA) was used at all times. HPLC-grade acetonitrile and formic acid, ammonium bicarbonate, iodoacetamide, dithiothreitol, sequencing-grade modified trypsin, and protease-inhibitor PMSF (phenylmethanesulfonyl fluoride) were purchased from Sigma-Aldrich (St. Louis, MO, USA).

#### 1.3.3 CSF collection

CSF samples were collected by lumbar puncture from patients who received spinal anesthesia before non-neurological operations at Beijing Tiantan Hospital. These patients were checked by an independent medical doctor to rule out neurological diseases and recent medication use. Following collection, a subsample of each CSF sample was sent to a clinical laboratory for routine CSF diagnostics. The remaining sample was immediately centrifuged for 10 min at 2500×*g* to remove cellular components and subsequently aliquoted and stored at −80 °C for further analysis. A total of 14 samples from 14 individuals (7 women and 7 men, aged 24–55 years, with a median age of 28 years) were selected and subjected to quantitation by the Bradford method [4]. Equal protein amounts from 14 CSF samples were mixed, resulting in the pooled CSF sample for the proteomic analyses. All selected samples had normal clinical laboratory values with respect to microbiology, chemistry, and cell counts. Approval for this study was obtained from our institutional review boards in accordance with ethical regulations.

#### 1.3.4 Immunoaffinity depletion of 14 high-abundance proteins

The pooled CSF sample, which contained approximately 1 mg of CSF protein, was depleted of 14 high-abundance proteins (albumin, IgG, α1-antitrypsin, IgA, IgM, transferrin, haptoglobin, α_1_-acid glycoprotein, α_2_-macroglobulin, apolipoprotein A-I, apolipoprotein A-II, fibrinogen, complement C3, and transthyretin) using a 4.6×50 mm Human 14 affinity LC column (Agilent, St. Louis, MO, USA) with a Waters HPLC system (Milford, MA, USA). The separations were performed according to the manufacturer׳s instructions regarding column usage and loading capacity. The flow-through protein sample and bound protein sample were collected separately. The two samples and the non-depleted CSF sample (containing the original proteins) were subjected to the sample handling and analysis procedures described below.

#### 1.3.5 Protein digestion

A filter-aided sample preparation method [3] was used to digest the proteins in the three samples (each contained 100 µg of CSF protein). Briefly, the proteins were reduced with 20 mM DTT at 95 °C for 3–5 min and washed once with 8 M urea on a 10 kDa filter at 14,000×*g* for 40 min. The samples were then alkylated with 55 mM iodoacetamide for 30 min in darkness and washed twice with 8 M urea. Next, the proteins were washed with 50 mM ammonium bicarbonate once and digested with trypsin (1 μg/50 μg protein) overnight at 37 °C. After digestion, the three peptide mixtures derived from the flow-through, the bound sample and the non-depleted CSF sample were desalted on a Waters Oasis C18 solid-phase extraction column and lyophilized for HPLC separation.

#### 1.3.6 High-pH HPLC separation

The three lyophilized peptide mixtures were fractionated with a high-pH RPLC column from Waters (4.6 mm×250 mm, C18, 3 μm). Each peptide mixture was loaded onto the column in buffer A2 (H_2_O, pH=10). The elution gradient was 5–30% buffer B2 (90% ACN, pH=10; flow rate, 1 mL/min) for 60 min. The eluted peptides were collected as one fraction per minute, and the 60 fractions collected were re-suspended in 0.1% formic acid and pooled into 30 fractions. A total of 90 fractions produced from the three peptide mixtures were analyzed by LC-MS/MS.

#### 1.3.7 LC-MS/MS

Each fraction was analyzed with a reverse-phase C18 self-packed capillary LC column (75 μm×100 mm, 3 μm). The elution gradient was 5–30% buffer B1 (0.1% formic acid, 99.9% ACN; flow rate, 0.3 μL/min) for 40 min. A TripleTOF 5600 mass spectrometer was used to analyze the fractions. The MS data were acquired using the high-sensitivity mode with the following parameters: 30 data-dependent MS/MS scans per full scan, full scans acquired at a resolution of 40,000 and MS/MS scans at a resolution of 20,000, rolling collision energy, charge state screening (including precursors with charge states of +2–+4), dynamic exclusion (exclusion duration 15 s), MS/MS scan range of 100–1800 m/z, and a scan time of 100 ms.

#### 1.3.8 Database search

The MS/MS spectra were searched against the Swiss-Prot human database from the UniProt website (www.uniprot.org) using Mascot software, version 2.3.02 (Matrix Science, UK). Trypsin cleavage specificity was set with a maximum number of allowed missed cleavages of two. Carbamidomethylation (C) was set as a fixed modification. The searches were performed using a peptide and product ion tolerance of 0.05 Da. The resulting dataset was further filtered using the decoy database method in Scaffold (v 4.3.2).

#### 1.3.9 Intensity-based absolute quantification (iBAQ) of proteins

Protein abundances were estimated using the iBAQ algorithm [2]. The detailed protocol is provided below:

(1) The protein intensities were first computed by Progenesis LC-MS (v2.6, Nonlinear Dynamics, UK) as the sum of all identified peptide intensities (maximum peak intensities of the peptide elution profile, including all peaks in the isotope cluster).
(2) The protein intensities were then divided by the number of theoretically observable peptides (calculated by in-silico protein digestion; all fully tryptic peptides between 6 and 30 amino acids were counted).
(3) The resulting intensities were iBAQ values, which are shown as “Absolute iBAQ intensities” in column K of Supplementary Table 2.
(4) The relative iBAQ intensities (in column L of Supplementary Table 2) were computed by dividing the absolute iBAQ intensities by the sum of all absolute iBAQ intensities.
(5) The relative iBAQ intensities were applied to estimate the relative protein abundances (the proportions of protein amounts to total CSF protein amount).
(6) The protein abundances (or concentrations) in CSF were finally calculated by multiplying the relative iBAQ intensities by 0.3 (the protein concentration of the pooled CSF sample).

#### 1.3.10 Protein networks and functional analysis

All identified CSF proteins were subjected to network and functional analyses using ingenuity pathway analysis (IPA) version 9.0 (http://www.ingenuity.com) and the PANTHER classification system (http://www.pantherdb.org/genes/batchIdSearch.jsp) [5].
